# Keeping in balance on the multimorbidity tightrope: A narrative analysis of older patients’ experiences of living with and managing multimorbidity

**DOI:** 10.1016/j.socscimed.2021.114532

**Published:** 2022-01

**Authors:** Nina Fudge, Deborah Swinglehurst

**Affiliations:** Wolfson Institute of Population Health, Queen Mary University of London, London, UK

**Keywords:** Narrative, Multimorbidity, Self-management, Chronic disease management, Primary health care, Bakhtin, Flourishing, England

## Abstract

Primary care management of patients with multimorbidity in the UK is underpinned by clinical guidelines, quality standards and measurable targets which govern practices of risk management and disease control. There is concern that standardised approaches may not always be appropriate for older patients living with multimorbidity.

Using a narrative approach, we elicited the voices of older people living with multiple conditions in order to rethink chronicity, and consider what their accounts might mean for reconfiguring care practices. Within an ethnographic study of multimorbidity and polypharmacy, we conducted in-depth interviews, based on the Biographical Narrative Interpretive Method, with 24 participants aged 65 to 94. Participants were recruited from three general practices in England. All had two or more chronic conditions and were prescribed ten or more medicines.

Our analysis draws on Bakhtinian theory, tracing the multiple ways in which participants voiced living with multimorbidity. In this paper, we focus on ‘keeping in balance’ which emerged as a key meta-conceptualisation across our dataset. Adopting the metaphor of the ‘multimorbidity tightrope’ we explore the precarity of patients' experiences and show their struggle to create coherence from within a deeply ambiguous living situation. We consider how and to what extent participants' narrative constructions co-opt or resist normative biomedical framings of multimorbidity. Our analysis foregrounds the complex ways in which patients' voices and values may sometimes be at odds with those promoted within professional guidelines. Narrative approaches may offer significant potential for reorienting healthcare towards enabling patients to live a flourishing life, even when facing significant adversity.

## Highlights

• A voice-centred analytical method illuminates participants' multivocal narratives.
• The ‘multimorbidity tightrope’ illustrates the precarity of patients' experiences.
• Managing multimorbidity requires careful balance in order to flourish.
• Participants both co-opted and resisted biomedical framings of multimorbidity.
• I-poems present the contradictory nature of participants' narratives.

## 1 Introduction

*“Patients facing the discomforts and demands of chronic illness struggle to maintain a productive, hopeful life. The effective management of chronic illness requires that they receive appropriate clinical care while they and their families appropriately cope with the illness and its therapies.”*Wagner et al., 1996. p.512

Wagner et al.’s (1996) classic text on the organisation of care for patients with chronic illness represented an important turning point for primary care. Modelled around proactive planned care, self-management, and supportive information systems, it called for a shift towards greater integration of evidence-based guidelines and intentional foregrounding of the ‘less urgent, but nevertheless predictable*’* needs of patients with chronic illness within a practice which had hitherto focused primarily on patients' acute medical needs. In particular, Wagner et al. were critical of the culture and structure of medical practice, arguing for radical ‘practice redesign’ to overcome caregivers' inability to meet the needs of chronically ill patients.

Within the last 25 years, there have been substantial demographic and organisational changes. Patients with chronic diseases are routinely monitored and managed in primary care as primary care teams strive to integrate care around the patient (Mercer et al., 2016). Formal programmes such as the Quality and Outcomes Framework (QOF) incentivise delivery of anticipatory care for patients with chronic diseases. Following its introduction in 2004, QOF became ‘business-as-usual’ relatively quickly, partly because it extended and accelerated trends in working practices that were already emerging (McDonald et al., 2013). In parallel the shift towards electronic patient records has enabled highly structured approaches, with evidence-based guidelines and protocols inscribed into computerised templates (Swinglehurst et al., 2012). Predictable aspects of chronic disease management are typically delegated to nurses with advanced training in particular disease areas, such as diabetes or asthma.

When considered in light of Wagner's call to action, this reconfiguration of care to deliver new evidence, new technologies and novel skill-mix has succeeded in ensuring patients with ‘less urgent’, more chronic needs have been prioritised. However, significant problems remain regarding *how* they have been prioritised. Critics of the ‘scientific-bureaucratic’ approach drew attention to the uncomfortable knot that policymakers had tied between ‘quality of care’, ‘pay for performance’ and quantifiable metrics, and their unintended consequences on notions of trust, professionalism and care practices (Checkland et al., 2007; Gillam et al., 2012; Harrison and Wood, 2000).

More recently, attention has turned towards the challenges of caring for older patients with multiple long-term conditions within a healthcare system oriented to single-disease care (Guthrie et al., 2011; Mercer et al., 2016). Strict adherence to multiple disease guidelines combined with policy-driven efforts at ‘self-management’ (Department of Health, 2007; NHS, 2019) can overwhelm a person's capacity to cope with a life already challenged by multimorbidity (Eaton et al., 2015; Mercer et al., 2016, Swinglehurst et al., 2012), leading to an excessive, sometimes dangerous ‘burden of treatment’ (Harrison and Wood, 2000, May et al., 2014, Swinglehurst and Hjörleifsson, 2018). There is concern regarding inappropriate extrapolation of evidence derived from clinical trials involving younger patients who do not suffer the multiple morbidities encountered in older populations. Critics also raise moral concerns. Using preventive, risk-reducing treatments in older age (as advocated in single-disease guidelines) may de-select one cause of death in favour of another without a patient's consent, whilst simultaneously ‘cast(ing) a shadow over a currently healthy life by the threat of disease’ (Mangin et al., 2007). Privileging risk-reduction may, in the context of multiple age-related risks, contribute to escalating polypharmacy, paradoxically exposing patients to well-recognised, but much less clearly articulated risks of ‘too much medicine’ (Swinglehurst and Hjörleifsson, 2018).

The normative, standardised approach to care of multimorbid patients is also problematic for general practitioners (GPs). A recent meta-ethnography highlighted four concerns, contributing to GPs’ sense of professional isolation: disorganisation and fragmentation of healthcare; inadequacy of guidelines and evidenced-based medicine; challenge of delivering patient-centred care within a system focused on specialism; challenges in shared decision-making (Sinnott et al., 2013). The results suggest tension between a professional commitment to subjectivity and personhood within a system logically committed to a more objective population-based approach to care (McDonald et al., 2013). This tension may be particularly pronounced when managing older patients, since inter-individual variability in health, disease and disability increases with ageing; it becomes harder to draw generalised conclusions about how best to manage chronicity in particular individuals (Nelson and Dannefer, 1992). The best care for older people with multimorbidity is not, it turns out, that predictable after all.

Researchers have responded to these shortcomings by investigating the potential of a ‘patient-centred’ approach to care - a ‘3D intervention’ incorporating dimensions of ‘health, depression and drugs’ reflecting international consensus on optimal management of patients with multimorbidity (Salisbury et al., 2018). This large, rigorously conducted, cluster-randomised trial found no difference in quality-of-life measures between those receiving usual care and those receiving the intervention (a six-monthly multi-disciplinary appointment replacing several disease-focused reviews). The researchers suggest the negative result may indicate that the causal model underlying the international consensus (which informed intervention design) is flawed. They question the assumption that better patient-centred healthcare for patients with multimorbidity will result in better health and wellbeing (Salisbury et al., 2018). Although this study sought to embrace the multiple interactions between patients' diseases and treatments, the underpinning logic informing the intervention, trial design and primary outcome measure does not represent a radical departure from the positivist model of technical rationality. Patients with multimorbidity may present what Schön (1983) refers to as a dilemma of ‘rigor or relevance’; a dilemma typical of the so-called ‘swampy lowland’ where some of the greatest human concerns lie beyond the reach of ‘technical’ solutions emerging from the dominant positivist epistemology of medical practice.

A significant body of anthropological and sociological research has chronicled the everyday practices of people living with medical conditions (Langstrup, 2013; McCoy, 2009; Mattingly et al., 2011). Recent social science scholarship frames chronic disease in terms of ‘vitality’ (Bates, 2018; Wahlberg, 2018), and ‘chronic living’ (Wahlberg et al.). Also circulating within biomedical, public health and popular cultural contexts is the discourse of successful ageing – positioning individuals as obliged to remain productive, active and healthy by using medicines and other technologies (Kaufman, 2015; Lamb, 2014). However, studies such as the 3-D study may suggest a need to readjust expectations of what medicine can achieve with ageing bodies. As Kaufman (2015) argues: ‘Although few appear to have foreseen our current predicament, it was inevitable that medicine would collide with age’. These studies may also provoke more radical rethinking of what constitutes genuine patient-centred care in this group – a shift which Jocelyn Cornwell argues might represent ‘the most disruptive innovation in healthcare’ (Dowrick et al., 2016, Swinglehurst et al., 2013). Patient-centred approaches continue to position the patient as object of the medical gaze, or participant in rational, biomedically-informed decision-making. A more person-centred approach might invite greater opportunity for patients to share their ongoing experience of suffering in the context of multimorbidity.

In this paper we report findings of a voice-centred, relational method of analysing in-depth narrative interviews involving older patients affected by multimorbidity and prescribed ten or more medicines. Tracing voices of co-option and resistance, we present the balancing act that patients endure to keep going in the face of ageing and chronicity as they ‘manage the multimorbidity tightrope’. First, we introduce the concept of ‘voice’.

### 1.1 Voices of struggle and resistance in the face of chronicity: the multimorbidity struggle

To investigate how patients live with multimorbidity, we draw on the theoretical work of Bakhtin. Bakhtin (1984), a Russian literary critic, conceptualised ‘voice’ as the dialogically constituted ‘speaking consciousness’, an expression of the ‘ideological becoming of a human being’ through assimilating and appropriating the words of others and populating them with one's own ‘evaluative accent’. Under this view, language is not simply a neutral linguistic resource representing experience but a dynamic process of constituting personhood or ‘becoming’, (re)articulating particular values and viewpoints. Language originates in social interactions and struggle; voices are social, dynamic and changing (Maybin, 2001; Sørenssen, 2018).

One particular struggle is that which plays out between what Bakhtin calls ‘authoritative’ and ‘internally persuasive’ discourse and the degree to which one voice has the authority to come into contact with and interanimate another (Wertsch, 2001). Authoritative discourses are relatively fixed, inflexible discourses (e.g. religious dogma, scientific ‘truth’); these are in tension with inwardly persuasive discourses which remain open, provisional and flexible (Maybin, 2001). Amongst patients with multimorbidity we encounter an authoritative biomedical discourse which emphasises managing chronic illness through clinical investigations, monitoring and medicines, with patients adhering both to medicines and ‘self-management’. This discourse pervades policy, professional documents and practice, and was readily taken up and articulated by our participants. Patients also voiced divergent ideas based on their daily experience of living with multimorbidity. What emerges are expressions which illustrate the polyphonic nature of voice: several voices, sometimes conflicting and contradictory, interwoven within single utterances, as speakers populate their accounts with their own evaluative accents (Sørenssen, 2018). Close study of the relationships between these voices offers a window into the perspectives, ideologies and tensions inherent within wider society (Brown, 1999; Maybin, 2013; Sørenssen, 2018).

Methodologically, we draw on the work of Brown (1999) and other scholars who have theorised and prioritised voice (Chadwick, 2020; Maybin, 2013; Sørenssen, 2018). Chadwick (2020) argues that whilst qualitative research often aspires to ‘give voice’ to participants, it often fails to acknowledge the careful, active listening required to work critically with voices thus avoiding simplifying participants' lives into ‘disembodied texts, discourses and themes’. Prioritising voice deliberately positions the research participant as expert by experience, and engages the researcher as curious listener, providing scope for surprise and discovery (Gilligan and Eddy, 2017; Simandan, 2020). Chadwick advocates for analytical methods that preserve the messy, contradictory, heterogeneous, relational nature of voice including practicing embodied listening and using tools such as the Listening Guide where voice and self are theorised as multivocal (Brown and Gilligan, 1991; Chadwick, 2020). Adopting this approach, our analysis involved tracing the multiple, contested and contradictory ways in which participants accounted for, and constructed their multivocal ‘selves’ in response to a single question.

## 2 Methods

We conducted a longitudinal in-depth ethnographic study of older adults experiencing multimorbidity and polypharmacy (Swinglehurst and Fudge, 2019). Between 2018 and 2020 we followed 24 people (13 women, 11 men, aged 65–94) with multiple long-term conditions, prescribed ten or more items of medication. We recruited participants from three GP practices - two urban practices and one suburban practice in South-East England. We engaged in: interviews; regular home visits and telephone conversations; accompanying visits to GP and outpatient appointments.

Participants had a range of common chronic conditions: hypertension, diabetes mellitus, COPD, atrial fibrillation, depression, anxiety, coronary heart disease, congestive heart failure, asthma, osteoarthritis, chronic kidney disease, diverticular disease, blood disorders. In addition, some were survivors of cancers or had rarer conditions (e.g. multiple sclerosis, narcolepsy).

In this paper, we focus primarily on interviews conducted using the Biographical Narrative Interpretive Method (BNIM). We elicited detailed narratives based on participants' responses to a single interview question and its elaboration, seeking to understand the person on their terms (Rosenthal, 2007; Wengraf, 2001). Our opening question was ‘Please tell me the story of your life since you were first advised to take medicines’. Interviewees started wherever they liked and included the people, experiences, and events important to them. Participants spoke uninterrupted, until their story was complete. After a 10–15 min break, we asked further questions using ‘cue phrases’ (participants' own words) noted during the opening narrative, focusing on events and experiences in the order they were initially presented. Interviews (mean 63 min) were in participants' homes, audio-recorded and transcribed verbatim.

We used sound recordings and interview transcripts in parallel in our analysis, alongside ethnographic fieldnotes. The Listening Guide involves three steps and multiple ‘listenings’ (Brown and Gilligan, 1991; Gilligan and Eddy, 2017):

1. Multiple listenings to identify: plot; characters; stories told; emotional hotspots; salient images; metaphors; major and minor themes. This includes noticing ruptures in the narrative, who and what is missing, and making reflexive notes on the listener response.
2. Construct ‘I-poems’ from each transcript by highlighting all the first person utterances, applying each use of ‘I’ to a new line in a poem and organising these into stanzas.
3. Identify the different (contrapuntal) voices which intersect, interact, and coexist, focusing on the quality of the participant's voice, including what is said (and said differently) and what is not said or silenced.

We shared detailed reflexive analytical memos at each step, discussing them to refine our understanding of the voices across the dataset, to consider what surprised us and to develop a meta-level conceptualisation of our participants’ voices.

Our study has National Health Service (NHS) ethics approval (IRAS project ID: 205517; REC reference 16/YH/0462). Our consent process involved: two home visits per participant; time for participants to read an information sheet between visits and discuss their participation with their family/friends/carers. All participants had capacity to consent and signed written consent forms. Names reported in this paper are pseudonyms.

## 3 Results

### 3.1 Managing the multimorbidity tightrope: the struggle for balance

‘Keeping in balance’ was a key meta-level conceptualisation of the voices we encountered in our analysis, and resonates with our analysis of our wider ethnographic dataset and with the work of Mol (2008) who studied care practices and self-care in a hospital diabetes clinic. Mol describes the work of caring as a practice of seeking balance and supporting moderation: ‘a matter of attending to the balances inside, and the flows between a fragile body and its intricate surroundings’, a matter of encouraging patients to find a middle ground between taking better care of themselves and ‘trying too hard to fight the unpredictability that is inherent in a life with chronic disease’ (Mol, 2008:25).

GPs in our study talked about their reluctance to ‘*upset the status quo*’ by changing patients' medication, especially if test results indicated the patient was ‘*in balance*’ and patients made no demands for change. Efforts to reduce or change medication involved cautious, carefully negotiated and usually very modest alterations. Concern not to upset the *status quo* was shared by some of our patient participants. For example, Rita didn't want her lithium ‘*messed with*’ even though she did not think she had ever needed it and repeatedly questioned the indication for it. Elaine, who described her health and medicines as ‘*all stable and under control*’ expressed uncertainty at a pharmacist's suggestion to drop aspirin from her daily pill count as she had been ‘*taking it for donkey's years*'. Adding ‘*I don't know … I'm still thinking*’, she hesitantly explained that she had been ‘*set all these medicines by the cardiologist … and they don't think I should come off them.*’ It was not only her pharmacists' voice she needed to consider but the more distal, historical voice of her specialist.

Clinicians' expressions of the concept of balance often (though not always) focussed on rather narrow interpretations of disease metrics, or involved general enquiries into patients' specific conditions or body parts. However, the reach of the ‘balancing act’ articulated by our patient participants was much more extensive. Participants valued their clinicians' input into their care and readily reproduced the metrics and objectives that their clinicians were primarily concerned with, but they extended the scope of their balancing act to incorporate wider social obligations and personal desires. A ‘deontic voice’ (doing as they *should* or *ought to*, according to the medical ‘rules’) often co-existed alongside an ‘agentic voice’ (a desire for agency to live life and manage multimorbidity as they wanted to - which sometimes meant managing less or managing differently to the rules).

Mishler (1984) famously contrasted the ‘voice of medicine’ and the ‘voice of the lifeworld’, arguing that the former was capable of suppressing the latter, stripping meaning and context from patients' accounts of their suffering and contributing to ineffective care. Silverman (1987) argues that medical discourses have now become so readily embedded in modern society that the strict distinction between the voice of medicine and the voice of the lifeworld is problematic. Furthermore, the voice of medicine is not inherently bad. Indeed Barry et al. (2001) showed that consultations in which both clinician and patient readily adopted the voice of medicine could proceed very successfully, though this mode of interaction lent itself most readily to relatively straightforward consultations focused on acute physical problems. In contrast, they found that the poorest outcomes occurred when patients used the voice of the lifeworld, but doctors persisted in their use of the voice of medicine, thus ignoring or ‘blocking’ patients' efforts to have their lifeworld concerns addressed.

In the following sections we show how our participants with multiple chronic physical problems voiced what we conceptualise as the ‘multimorbidity tightrope’, adopting not a single ‘voice of the lifeworld’ but multiple intersecting voices. This tightrope metaphor captures the precarity of their situation, the risks and dangers involved, the imperative to ‘keep going’ amidst the struggle, and the courage that may be needed to resist authoritative discourses as participants seek to live a flourishing life that enables independence in the context of increasing dependency (Toon, 2014).

### 3.2 Balancing the demands of the clinic

Participants valued their clinicians and regarded relationships with their GP and other staff at their local surgery as important, despite reporting numerous frustrations: lack of GP appointments; time-limited appointments; difficulty securing home visits; the need for multiple attendances. Specialists, who they typically held in higher esteem, focused only on their specialisms, leaving patients perplexed as to whether anyone was able or willing to care for them as a whole. Patients worked hard to balance the demands of their professionals and their medicines into their lives, acknowledging both their dependence on all the different parts of the healthcare system and their desire to be free of this dependence. Participants were deeply ambivalent about their situation. Kay, 74-year old, housebound, and prescribed 30 separate items of medications oscillated between describing her dosette box as ‘*beautiful*’ and saying ‘*I have a great big bag of medicines which I'd like to throw into the sea*’ as she questioned whether her medicines, which she took dutifully, were doing any good. The following quote, replete with an awkward double negative (described as ‘logic’), captures her ambivalence and points to her ‘*sick*’ experience being at least partially treatment-related:

You go through all these stages … er … you know you shouldn't, that's the thing though. You know you shouldn't not take them, that's logic; you should take them but you don't want to take them, you're sick of it.

Rita, also housebound, traces her descent into a lifetime of multimorbidity to a street assault; she was ‘*grabbed by the throat*’ and held up at gunpoint outside her doctor's surgery. Secure behind the locked wrought iron gate across her front door, she shares her story of the work she must do to stay in balance. Displaying considerable medical knowledge about her chronic conditions, she presents herself as proactive in asking the doctor for what she wants and adopting a responsible self-management role. She incorporates the language of ‘*levels*’ in her interview, and wants to ensure that her blood measurements are ‘*where they should be, the level, you know*’. But she would rather *not* have to organise hospital transport for monthly one-day round trips to the hospital for blood tests to support this. In the following I-poem we hear her navigate this tension (balancing demand with not being *too* demanding) in her efforts to assure her platelets are ‘*level*’. Of note, the elision (‘I'm quite … ‘) suggests she struggles to find a word to articulate this work:

I was diagnosed with thrombocythaemia

I'm on medication with that

I have to have regular blood tests

I told my doctor

I said “Rebecca could I have somebody round regularly, to take my blood?”

I take hydroxycarbamide

I've never read up on this thrombocythaemia - the COPD is enough for me to worry about

I know that your platelets and your red and white cells, they're all important

I'm quite …

I said to the GP “Could I have somebody round to take my blood regularly?”

I know my platelets need to be at a certain level, my red cells, white cells, haemoglobin, I think that comes into it

I got settled on my hydroxycarbamide

I know it's because they're so busy, nobody phones me up now

I haven't really phoned them up

I don't know why

I don't know, because they're so busy,

I know what it is you know?

I'm on the phone again

I hate having to

I hate it, you know, being a nuisance to the people, you know?

In the first stanza, Rita is assertive in her conviction to keep up with the tests, twice incorporating her request to her GP for regular home visits to enable this as reported speech. But by the end of her I-poem there is less certainty in her voice and a realisation that her housebound status, coupled with a stretched health service, makes following a blood testing regime difficult. She uses an extreme case formulation (‘*nobody phones me up now*’) to make her point (Pomerantz, 1986). Ultimately she is negotiating a delicate balance between being a proactive, self-managing patient, responding to the deontic voice of medicine (*I have to have [blood tests]; my platelets need to be at a certain level*) and ‘*being a nuisance*’.

In a similar acceptance of the limitations of medicine and likewise not wishing to bother the GP unnecessarily, Marco says:

So, I try to live with it. Sometimes there's nothing I can do about it, and me going to the doctors, there's nothing she can do about it, you know. What's she going to do? You know, she'll do, she'll send me for other tests; well, the tests, we already know the tests. So the best thing is (…) just leave it alone; if it gets bad, then I have to go and see her, but that's the thing.

His opening remark ‘*I try to live with it*’ conveys the struggle of living with chronicity. He uses two extreme case formulations (‘*there's nothing I can do about it*’; ‘*there's nothing she can do about it*’) followed by a rhetorical question: ‘*What's she going to do?*’ He suggests the GP will send him for tests, but ‘*we already know the tests*’. This narrow construct of the GP as ‘organiser of tests’ which may reflect one aspect of his experience of multimorbidity, communicates a message of futility around this response and its limited form of ‘knowing’. Only when things ‘*get bad*’ will he ‘*have to go*’ and see the doctor.

When talking about medical investigations participants used ‘fleshy’ voices (Chadwick, 2020) communicating embodied experiences of discomfort, sometimes expressed using metaphors of violence. Charles opened his biographical account with ‘*my first recollection of medical interference …* ’ and Marco used a shocking metaphor (‘*it's like rape*’) to describe the occasion when a consultant recommended an implantable defibrillator. In the following quote, Rita articulates a small act of resistance as she attempts, unsuccessfully, to negotiate an alternative way of providing blood for testing to the respiratory clinic that is less painful and less embarrassing:

They cut your ear like that (*she elaborates with a quick sweep of her hand next to her ear*) and obviously it bleeds, and then they take a sample, and they can tell what the oxygen in your blood is and that's the best place. Because I said to the lady, “Can you not do this anywhere else rather than my ear?” and she went, “No, that's the best place we do it from, and that will tell us what percentage of oxygen you've got in your blood.” I said, “Well that's marvellous, isn't it?” And then they stick a big plaster on it; it's all stuck to your hair and everything! You're in a right state; you know, when you go out, everybody is sort of looking at you, you know? Look at the state of this one!

Marco recounted the pain he experiences in his fingers and stomach from testing his blood and self-administering insulin to achieve balance in his blood glucose measurements, captured in this I-poem:

I've been seeing the diabetic specialist and the insulin goes up to 60ml, right?

I need to increase it

I have to take four shots: two in the morning, two at night.

I said, “No, I don't really want to take four shots”

I said, “You must have something that does more than 60!”

I'm taking the blood samples twice a day.

I'm taking the bloody insulin, the insulin I take in my stomach

I do get sore, and my fingers get sore, you know?

Marco oscillates between the deontic voice ‘*I need to increase it; I have to take four shots*’, in which the metaphor of ‘*shots*’ conveys pain and violence, and his agentic voice of resistance to the new insulin regimen: ‘*No I don't really want to take four shots*'. He asserts that the diabetes specialist must find a device that can accommodate the increased dose to save Marco from increasing the number of times he has to inject. Although in this I-poem we hear Marco say ‘*I'm taking the blood samples twice a day*’ we learn later in our fieldwork that due to the soreness from the finger prick tests he only does his glucose testing in the week running up to his diabetes check with the nurse. It's a balance.

In summary, these accounts show the multiple and contradictory voices that patients bring to their expressions of the work of meeting – and resisting - the demands of the clinic in their efforts to achieve balance in their fragile, precarious lives with multimorbidity.

### 3.3 Balancing the demands of life beyond the clinic

Beyond the clinic, our participants faced additional demands negotiating living as they ‘should’ and living as they ‘want to’ in the context of multimorbidity. The rules of the clinic carry over into their daily lives and are joined by other rules that govern daily experience, often reinforced by family members and social networks. Patients articulate a struggle to seek balance in engaging with the rules they live by, a delicate act of juggling risks, practicalities and social obligations.

For Iris, who has narcolepsy among other conditions, hanging out in the market with friends is important. She meets them almost every morning. The risk of unpredictable, uncontrollable narcoleptic episodes (her ‘*attacks*’) in which she suddenly falls asleep generates anxiety for her family. Family members come and go to her flat and she is rarely left alone; her grandson is almost always present when we visit. Her family have devised informal rules for her to live by (*‘they tell me “you must not do things alone”*’), but she has to resist these rules to maintain her sense of self. This I-poem is full of contradictions and contrapuntal voices:

I don't think life's changed really

I can't do the things that I used to do

I can't travel on my own

I can go to [local market]

I sit down on the bus I go asleep

I obviously don't

I'm travelling to like [large shopping centre]

I go on my own

I take a chance

I come back

I'm asleep

I end up through the tunnel

I realise I've passed my stop

I just get off and make my way back

I can't do the things I used to do

I can't go nowhere on my own now, like out

I was quite capable of going out

I go out, they do their nut

I don't rely on no-one

I say if it happens, it happens

I look at it

I've got to live with it

I've got no choice

Her opening line ‘*I don't think life's changed really*’ is immediately followed by two lines marking out what she cannot do, although it quickly becomes obvious that she does indeed travel on her own despite her family's reaction (‘*they do their nut*’). Iris has appropriated the voices of others – clinical staff, family members – to such an extent that she absorbs them into the first person: ‘*I can't travel on my own*’. Yet she retains her independence and goes out on her own – a fact she repeats three times in this extract. Life for Iris *has* changed in practice, but she holds on to her independence and will ‘*take the chance*’ to go out on her own, locally at least, and ‘*if it happens, it happens*’.

Negotiating the perils of the ‘multimorbidity tightrope’ was an ongoing and deliberate activity for participants as they constantly adopted strategies which enabled them to maintain, as far as possible, their independence and live the life they wanted in the context of increasing dependency. Marco accepts a walking stick, a visible signifier of his mobility limitations, as this enables him to get out and about and make regular trips to his local pharmacy to pick up his medicines:

I was going to get my medication in the other chemist

I'd no stick at the time

I got the most terrific, terrific pain in my leg that I've ever had;

I was half way there and half way here.

I persisted

I bought a stick there

I said, no, to leave it.

I had a drop of water

I bought a stick there.

I don't go out without my stick

I got that stick

I was leaning on it

I don't think I would have got back home on my own, you see.

I get, I get twitches in me leg

I do actually need the stick

[in the home there's many things that] I can hold onto,

[but that stick] I never go out without it.

Throughout the I-poem he builds an increasing sense of his reliance on his stick (I don't go out without my stick; I do actually need the stick; I never go out without it), However, he rejects the pharmacist's offer to organise his medicines into a dosette box and have them delivered direct to his door, as he regards maintaining purposeful independent control over how he organises his medicines as a signifier of ‘*capability*’ and, by contrast with his stick, says he doesn't ‘*need*’ it. He speaks somewhat disparagingly about people in adjacent flats who he sees receiving such deliveries when he thinks they are ‘*capable*’:

I don't have mine delivered, on purpose.

I don't have my medication already done, on purpose …

I don't have it so it still gives me something in life to go and get my medication

I mean, sometimes you're offered a service, but it doesn't mean that you really need that service, you know what I mean?

I can do it myself

I can do it myself

In contrast, Sydney, barely comments on the dosette box his pharmacy started to deliver without warning or discussion: ‘*Now they've put me on, they've agreed with the doctor, to give me a week's supply which they put in a box.*’ Sydney's narrative is broadly one in which he presents himself as accepting of, and compliant with medical advice (‘*I do as I'm told actually*’). However, he strongly resists an offer of a new battery operated oxygen machine to replace the oxygen bottles he has delivered weekly. The recommended machine requires charging before use and Sydney, who regularly participates in community groups, envisages that this will greatly inhibit his ability to go out when he wants, without notice. In the following I-poem this voice of resistance sits alongside his evaluation of what is important to him as he finds ways of managing his COPD into his life. Of note, he has successfully resisted this new machine secure in the knowledge that the physiotherapist has left open the option for him to change his mind and ‘*reach*’ her.

I explained to the physiotherapist

I wasn't going to sit on that machine for 16 hours a day

I like to go out

I was going to two lunch clubs for a start off

I was on the machine for 16 hours

I went out

I took an oxygen bottle.

I said no

I was used to the oxygen bottle,

I didn't have to carry it when I went out

I have a scooter

I would stick to the oxygen bottle

I knew [it]

I was used to [it]

[if] I changed my mind,

I could always get in touch with her

I can reach her

[if] I want to.

Zac, almost 90, presents himself as an independent, educated and capable man, determined to overcome adversity. His quest for independence is not new, but he guards it fiercely, situating it within a poignant biographical account of his experience of racial discrimination since he moved to London in the 1960s as part of the Windrush generation. This is illustrated in the following quote and I-poem, both of which show his struggle to have his voice heard and his need to secure supportive relationships to enable this:

It wasn't easy finding any place; as you probably heard, when you go you see a sign for a room or flat to let, and as soon as you get there, sometimes they look out, open the door and see you're a black person, and just close the door. Or the sign would say, “No blacks, no Irish and no dogs.” And uh … It puts you off, whoever you are.

I'm not kidding you

I'll never forget this

I went back with another friend of mine

I went back with this white person and he was able to explain who I was and what my education was

I got the flat because he was able to talk and explain everything

I first went, they only saw me

I went back with this other white guy

I got it.

I'd been there for six months or so, [the landlord] and I became quite close.

I stayed there for years.

Negotiating the balance between independence and dependence in the context of valuing and preserving trusted relationships features strongly throughout Zac's narrative. The unsafe neighbourhood where he lives limits his visits to church after dark, especially since he was mugged on his way to an evening meeting, but despite offers from church friends to drive him home he is reluctant to accept for fear of imposing ‘*too much*’ on them:

I feel

I don't want to be constantly asking her to do that.

I know she will do it,

I don't like to impose on people too much.

Like Marco, Zac has resisted having a dosette box whilst accepting that managing his medicines himself may not always be possible. For as long as it is possible, ‘*I'm happy*’:

I'm lucky enough that

I don't want dosette boxes

I am able to choose and put my medication together

I have exactly what

I should be taking

I try as far as I can

I might not be always able to do it

I'm able to put mine together

I'm happy

In this section we have seen how patients with multimorbidity juggle their clinicians’ demands for tests, monitoring and balance in disease metrics with a wider desire for balance in their life-as-lived, striving to pursue their personal values and live purposeful, flourishing lives. This takes in activities such as meeting friends, attending church, staying active, principles such as self-reliance, and orientations towards living life such as managing risk or desire for spontaneity.

## 4 Discussion

Our analysis shows our participants both co-opting and resisting biomedical framings of multimorbidity. Although ‘authoritative’ biomedical discourses are readily articulated, we also highlight the ‘internally persuasive discourses’ which jostle alongside. Patients work hard to find a balance between what is desirable from a biomedical perspective (a ‘deontic’ voice governing what they *should* do) with what is possible, desirable and achievable in daily life. The tension between these poles is especially stark for patients with ageing, multimorbid bodies. They encounter a health system organised around a ‘single disease’ model which focuses primarily on seeking ‘balance’ in bodily parameters rather than ‘balance’ in *living with chronicity*. Patients struggle to identify who can help them, and whether and how they might do so. They express profound ambivalence about their situation and their experience of care as they struggle to pursue the biomedical ideal whilst just ‘getting on’ with life. What emerges is necessarily a compromise. In the end, getting on with life is priority.

Striving for ‘balance’ incorporates not only the demands of the clinic (the pursuit of bodily homeostasis, acceptable ‘levels’ of blood glucose or platelets for example), but reconfiguring how to ‘do’ living in the context of chronicity. It is inevitable that patients must adapt their lives to accommodate their multimorbidities. The challenge is to find a balance that enables them to pursue what they value most, honouring their obligations to self and others in the context of shifting dynamics and dependencies. Patients are strategic about what help they seek, accept or resist. This is a necessary aspect of honouring their own independence whilst ensuring that important relationships endure. It does not do to be a nuisance or a ‘bother’ to others. It is a balance.

There have been calls to expand the predominantly biomedical conception of multimorbidity to encompass a broader understanding of the complexity of living with multimorbidity for older adults (Coventry et al., 2015). Without such expansion, there is concern that health services might inadvertently sustain the mismatch between the needs of patients and the services they receive, and (paradoxically) fail to support agency and purposeful action in the multimorbidity context (Northwood et al., 2018; Ploeg et al., 2017). Even sophisticated approaches to delivering patient-centred care, such as the 3-D intervention (Salisbury et al., 2018) may fall short of embracing chronicity in its full complexity.

### 4.1 Rethinking chronicity: supporting flourishing in the face of multimorbidity

Our analytic approach encourages a focus on the struggle that plays out in language as participants grapple with constituting personhood and ‘becoming’ in the face of multimorbidity and ageing. It illuminates in nuanced detail what Wagner refers to as the ‘*struggle to maintain a productive hopeful life*’ (Wagner et al., 1996). It also complicates the taken-for-granted understanding of language as simply representative of meaning, makes visible (or ‘hearable’) the inherent contradictions in meaning and shows the discursive work that patients do to create coherence out of complexity, contradiction and ambiguity (cf. Lee, 2019; Svensson, 2021). Arguably this approach not only opens a window into the *discomfort and demands of chronic illness* (Wagner et al., 1996) but witnesses them as they play out. We become sensitised to the potential of dialogue itself as an opportunity for participants to ‘become’, to make sense of contradictions and ambiguities within their lives. We discover that the activities of the clinic are fraught with their own potential for discomfort and demands. Even the offer of help from a friend has to be carefully weighed in the search for ‘balance’. The corollary is that the approach also reveals the possibility inherent in the clinical encounter as an occasion for ‘becoming’.

The Aristotelean concept of *eudaemonia* is helpful here. Translated as ‘flourishing’ it encompasses the notion that life is dynamic, and has a purpose and a shape - it is a narrative with a meaning (Toon, 2014). The notion of flourishing not only encompasses periods of growth, development and maturity, but also decay, and ultimately death. This concept has application throughout life and, as Toon argues, has important implications for healthcare. This may be especially true for patients like those in our study, who are seeking to live a meaningful life in the face of ageing and multiple, incurable conditions. A focus on flourishing rather than the ‘myth of cure’ (Heath, 1995) prioritises making meaning within the life narrative, on the dialogical construction (i.e. co-construction) of a purposeful self, or the ongoing process of ‘becoming’. There is no doubt that biomedicine can contribute to supporting patients to live flourishing lives; the readiness with which our participants take up and reproduce biomedical discourses is an expression of this understanding. However, our participants also express voices of resistance, including frustration regarding the perceived futility of what is sometimes offered. Above all, they articulate their need to prioritise getting on with life over strict adherence to the demands of the clinic. A commitment to enabling flourishing goes beyond - and sometimes challenges - the biomedical. It requires clinicians to engage in a very active form of listening, to be genuinely curious about what might help a patient to flourish in their lives, and a willingness to adapt care (to the extent that this is possible) to reflect this. As Toon argues, it may mean rejecting possible but probably futile medical interventions (Toon, 2014).

### 4.2 Reflections on the Listening Guide approach

As researchers using the Listening Guide approach we have the luxury of multiple listenings, of ‘slowing down’ narratives, and exercising curiosity without obligation to make clinical decisions. Attention to this degree of nuance is impossible within a clinical encounter. However, we believe our work points to a need for greater sensitivity to patients' unique context and a willingness to suspend - even temporarily - the demands of the structured chronic disease template. This may allow opportunity to embrace patients' ambivalence about their lives with multimorbidity, be surprised by their accounts, and re-orient the consultation towards supporting patients in co-constructing meaning. For example, we were surprised at Rita's account of being held at gunpoint, troubled by violent metaphors describing medical interventions, and intrigued by Marco's and Zac's fierce resistance of dosette boxes.

We recognise that stories are necessarily partial, highly situated and incomplete. Our research focussed on older people, and called on them to recall historical events (e.g. “*Do you remember that time particularly strongly?“*). This was sometimes a struggle. Sometimes this struggle may have reflected cognitive difficulties. It may also reflect Simandan's (2019) third and fourth epistemic gaps, namely the inevitable loss or distortion of information between its real-time witnessing and its recollection, and the gap between what is privately remembered and what is socially shared in an interview. However our analysis focused on a different struggle, the dialogical struggle between conflicting and contradictory voices as they were woven together into a coherent account. Critical attention to narratives may, by virtue of their partiality, enable researchers and practitioners to ‘*build meanings and bodies that have a chance for life*’ (Haraway, 1988).

### 4.3 Implications for policy and practice

Iona Heath has described the unique contribution that GPs can offer in helping patients to makes sense of what they are experiencing, as ‘witness to suffering’ and ‘interpreter of stories’, enabling patients to share their burden of suffering and have their experience validated (Heath, 1995). In particular, Heath points to an obligation on doctors to debate with patients the limitations as well as the possibilities of medicine (Dowrick, 2017; Heath, 1995; Toon, 1994). This may be especially important in light of the findings of studies such as the 3D-study (Salisbury et al., 2018), which casts further doubt on the strength and applicability of available scientific evidence to support good care in the context of older patients with multimorbidity.

Chronic disease management systems provide simplified models to tackle very complex process and practices. They are underpinned by a predominantly consequentialist ethical commitment which is endorsed by population wide programmes such as the Quality and Outcomes Framework. This orientation does not leave much space for the individual narrative, nor attention to flourishing in life in its broadest sense. Our findings support an intentional move away from normative approaches to chronic disease management; patients' voices and values may not always resonate with those recommended by chronic disease guidelines and prevailing ‘good’ practice. Supporting practitioners to foster ways of working that integrate the notion of flourishing into their professional toolkit might strengthen relationships with patients, offer opportunity for professional reflection and create greater scope for both patient and professional to make sense of the multimorbidity struggle. What is needed is a valuing of this kind of work in processes such as professional appraisals and evaluations of quality and innovation in professional practice (Toon, 2014; Rethinking Medicine, 2020). Arguably a shift of orientation towards supporting flourishing, rather than managing metrics, might also provide clinicians with a fresh, more vital approach to their practice and free them too from the seductive, but ultimately disappointing ‘myth of cure’ (Heath, 1995).

## Credit author statement

Nina Fudge: Methodology; Formal analysis; Investigation; Writing – original draft; Writing – review & editing; Project administration. Deborah Swinglehurst: Conceptualisation; Methodology; Formal analysis; Investigation; Writing – original draft; Writing – review & editing; Funding acquisition; Supervision.

## Funding statement

This article presents independent research funded by the National Institute for Health Research (NIHR) through a Clinician Scientist Award CS-2015-15-004 (DS). Additionally, this research was supported by the National Institute for Health Research ARC North Thames. The views expressed in this publication are those of the author(s) and not necessarily those of the National Institute for Health Research or the Department of Health and Social Care.
